# Chronic pain and fatigue in adults with congenital unilateral upper limb deficiency in Norway. A cross-sectional study

**DOI:** 10.1371/journal.pone.0190567

**Published:** 2018-01-03

**Authors:** Heidi Johansen, Trine Bathen, Liv Øinæs Andersen, Svend Rand-Hendriksen, Kristin Østlie

**Affiliations:** 1 TRS, National Resource Centre for Rare Disorders, Sunnaas Rehabilitation Hospital, Nesodden, Norway; 2 Institute of Clinical Medicine, Faculty of Medicine, University of Oslo, Oslo, Norway; 3 Department of Physical Medicine and Rehabilitation, Innlandet Hospital Trust, Ottestad, Norway; Washington University in Saint Louis School of Medicine, UNITED STATES

## Abstract

**Purpose:**

To describe Norwegian adults with congenital unilateral upper limb deficiency (CUULD) regarding self-reported chronic pain (intensity, locations, impact on daily life) and fatigue. Analyze associations between chronic pain and demographic/clinical factors and associations between fatigue and demographic/ clinical factors.

**Materials and methods:**

Cross-sectional study. In 2012, a postal questionnaire was sent to 186 persons with congenital limb deficiency, age ≥ 20 years. Seventy seven persons with CUULD responded and are included in this paper. The questionnaire included questions on demographic and clinical factors, chronic pain (Brief Pain Inventory, Standardized Nordic Questionnaire) and fatigue (Fatigue severity scale (FSS)).

**Results:**

Mean age was 42.7 (SD 16.0), 71% were women. Sixty tree % reported chronic pain, many had bilateral pain, most common pain locations were neck (78%) and shoulder/upper arm (78%). However, reported mean pain intensity (3.3 (SD 2.8)) and mean number of pain locations (3.0 (SD 2.5)) were moderate to low. Thirty seven persons reported that pain started in adult age (≥ 19 years). One third reported severe fatigue (FSS ≥ 5). Persons reporting cold sensitivity and severe fatigue were most likely to have chronic pain.

**Conclusions:**

Congenital upper limb deficiency increases the risk of self-reported pain in neck, shoulder/upper arm, cold sensitivity and severe fatigue. Pain, fatigue and cold sensitivity may individually affect function, and may together reinforce functional problems. This should be to taken into account when rehabilitation programs are developed. Further studies of more representative samples should be conducted to confirm our findings.

## Introduction

This paper presents data on chronic pain and fatigue in adults with congenital unilateral upper limb deficiency (CUULD) in Norway. The study sample is part of a congenital limb deficiency (CLD) population described in earlier papers [1, 2].

## Background

CUULD is the most frequent CLD, and is twice as common as lower limb deficiency [3, 4]. The incidence of CUULD has been reported as 5.25 per 10.000 live births [5]. Different nosologies and classification systems have been presented [3, 6]. In Norway, the ISO/ISPO classification of congenital limb deficiency is most frequently used [6]. In this classification, CLD`s are divided into transverse and longitudinal defects. In transverse CLD`s, all structures distal to a specific point of the limb are lacking. In longitudinal CLD`s, a bone or several bones are lacking partially or completely parallel to the long axis of the limb. In addition to the lacking parts of the limb, blood vessels, nerves and other structures in the remaining part of the limb may also be affected [7].

Missing the whole or parts of arm, hand and/ or fingers can cause problems in daily life as arms and hands are used for most activities. Deficiencies of fingers and hands may affect grip, the ability to lift and the range of reach. Persons with transverse upper limb deficiencies often totally lack grip, those with longitudinal deficiencies often have some kind of grip with reduced power and reach. Depending on the level of the deficiency, some persons with CUULD may benefit from the use of prostheses. Some prostheses are made for improving function, while others are used mainly for cosmetics [8,9]. Use of prostheses may also improve the participation in the society [8,9]. However, use of prostheses interferes with sensibility and the person`s natural movement patterns [1].

It has been proposed that persons with acquired- and CUULD may be prone to overuse problems [10,11]. In adults with acquired UULD an increased risk of self-reported musculoskeletal pain in the neck, upper back, shoulder and in the remaining arm was reported [12]. Prosthesis-use did not prevent pain [12]. Studies on mixed aquired- and CUULD samples have shown similar results [10,11,13]. The available data on prevalence, localisation, intensity and duration of chronic pain in adults with CUULD however, is limited, and it is uncertain whether they have the same prevalence and expression of chronic pain as persons with acquired UULD.

A longitudinal study of persons with unilateral below- elbow CLD [14], baseline examination at mean age 13 years and follow-up at mean age 37 years, found no increase in physical complaints, no scoliosis and no differences between prostheses-users and non- users in relation to physical complaints or scoliosis. Two studies on young adults with CUULD found no limitations in activity and participation, compared with the general population [15], and between mild and severe type of longitudinal radial deficiency [16].

However, in two earlier papers on the CUULD population, also described in the present study, we presented a significantly higher prevalence of chronic pain (63%) [1] and lower health related quality of life [2] than in the Norwegian general population (NGP). Health-related quality of life was most reduced in the physical health domain, and the greatest difference was found in the bodily pain scale [2].

Chronic pain is a complex phenomenon covering both physiological and psychosocial aspects [17]. Chronic pain is the main cause of long term sick-leave and disability in Norway [18–20]. In the NGP chronic pain is often widespread, generalized and associated with female gender, high age, low education level, receiving pension, psychological distress, chronic illness and low physical activity [18–20].

In other patient groups like Marfan syndrome, rheumatoid arthritis and poliomyelitis [21–23], fatigue has been found to be the factor most significantly associated with chronic pain. Fatigue is often defined as an “overwhelming sense of tiredness, lack of energy and feeling of exhaustion, mental, physical or both” [24]. Fatigue is prevalent in the general population [25], it is a common symptom in various chronic diseases, and has been found to be a major determinant of disability, which significantly influences quality of life and impairs people’s work ability [21, 26, 27].

Based on our clinical experience, persons with CUULD often report tiredness and/or pain. To our knowledge there has not been focus on fatigue as a phenomenon in this population. We therefore wanted to explore the aspects of chronic pain and fatigue in adults with CUULD.

The aims of this paper were to: 1) Provide a further description of self-reported chronic pain (intensity, locations, and impact on daily life), 2) Study the prevalence of severe fatigue, 3) Explore the associations between chronic pain and demographic and clinical factors and 4) Explore the associations between fatigue and demographic and clinical factors among adults with CUULD in Norway.

## Materials and methods

### Design and subjects

In this paper we used data on persons with CUULD from a cross-sectional, questionnaire-based study of adults with CLD. Potential participants for the CLD study were identified using the electronic database of the TRS National Resource Centre for Rare Disorders (TRS). Persons ≥ 20 years with a CLD were asked to participate. The exclusion criteria were lack of mastery of written Norwegian, syndactyly, polydactyly, and Poland syndrome without CLD. The inclusion process is described in detail in a previous paper [1].

TRS acts as a supplement to Norway’s public health services [1]. Persons in one of eight rare diagnostic groups (CLD, artrogryphosis multiplex congenita, Ehler-Danlos syndrome, Marfans syndrome, osteogenesis imperfecta, short stature and spina bifida / myelomeningocele, multiple ostochondromas) may contact TRS, without a referral. Upon contact, they agree to be registered in an electronic medical database.

### Questionnaire

A questionnaire was designed in cooperation with representatives from the Norwegian Limb Deficiency Association (NLDA). Variables included: Demographic factors; given syndrome-diagnoses; comorbidity (additional diagnoses); use of technical aids and/or prostheses; pain and fatigue (detailed below). Description of the CLD (side, type and level for the affected limb, including marking the deficiency on a figure) was used to classify the deficiencies as left/right, level and longitudinal/transverse. The classification was done in accordance to Day 1991[6]. The description of the deficiency was used to create a dichotomized variable “grip ability”: no grip = no, reduced grip/near to normal grip = yes. Cold sensitivity was investigated with the question: Do you experience to freeze easily on the affected limb? (yes/no).

Pain was measured in several ways, using questions adapted from the Brief Pain Inventory [28] and the Standardized Nordic Questionnaire [29, 30]. These instruments are shown to have satisfactory psychometric properties [28, 29, 30], are validated for the NGP [19, 20], and other patient groups [31], but not for limb deficiencies. The following variables were used: 1) Experience of persisting chronic musculoskeletal pain more than three months during the last year (yes/no). 2) Side of pain (right/left/both sides). 3) Self-reported pain intensity (PI) over the last seven days, assessed with an 11-point Numeric Pain Rating Scale (NPRS) with 0 = “no pain” and 10 = “pain as bad as it can be”. A pain score of 1–3 indicates “mild pain”, a score of 4–6 indicates “moderate pain” and a score of 7–10 indicates “severe pain” [32]. 4) Pain drawings: The participants were asked to mark their pain experience during the past two weeks on silhouettes of the human body (front and back). The locations of marked areas were scored using a modified procedure of the Brief Pain Inventory [28]: Each drawing was divided into 11 areas using a template (head, neck, shoulder/upper arm, elbow/forearm, wrist/hand, upper back, lower back, front chest, hip/thigh, knee/leg, ankle/foot), and each area scored as yes/no. Summing up the number of marked areas, we made a “number of pain location” (NPL) score. The NPL was then categorized into three groups: “Few pain locations” (0–3), “moderate pain locations” (4–6) and “many pain locations” (7–11) [19]. 5) Debut of present pain: “In childhood” (0–12 years), “during adolescence” (13–18 years), “in adulthood” (≥ 19 years). 6) Pain impact on participation (yes/no) in: “housekeeping”, “working”, “leisure activities”. 7) A free text description of self-reported factors reducing and increasing pain.

Fatigue was measured using the Fatigue Severity Scale (FSS), a nine-item questionnaire developed to measure fatigue intensity and the impact on daily functioning [33]. Each statement (e.g. “I am easily fatigued”) is rated on a 7- point response scale, ranging from 1 (completely disagree) to 7 (completely agree). A mean score is calculated for each person, range 1 to 7 [33]. Higher scores indicate higher levels of fatigue. FSS has been used in the NGP [25] and in different chronic diseases [24], and has been found valid and reliable [33]. To assess the prevalence of “severe fatigue” versus “no fatigue”, the following cut-off values were used: “No fatigue” = FSS mean score ≤4; “severe fatigue” = ≥5; and “borderline fatigue” mean score >4 and < 5 [21,25].

To measure health related quality of life (HRQOL) the MOS SF-36 version 2 was used [34]. The SF-36 consists of eight subscales, four mental scales: mental health, role functioning emotional, social functioning, vitality and four physical scales: general health, bodily pain, physical functioning and role functioning physical. Mean scores may be reported for each individual subscale and for two sum scores: mental component summary (MCS) and physical component summary (PCS). All subscales in SF-36 have 0–100 scales, 100 is best health status score [34]. The HRQOL in our CUULD sample is reported in detail in an earlier paper [2]. In the present paper, we used the physical functioning—and mental health scales to explore associations between pain and fatigue and physical functioning and mental health. The SF-36 sum scores were not appropriate to use as independent variables in the regression analyses (with pain and fatigue as dependent variables) because bodily pain and vitality scales are included in the sum scores.

### Data collection

The study has been approved by the Regional Ethics Committee for Medical and Health Research Ethics in Eastern Norway and by the Data Protection Officer at Oslo University Hospital (2012/805)

In October 2012, letters were sent to 186 adults with CLD, including a letter for written informed consent, the questionnaire and a prepaid, return-addressed envelope. A written reminder was sent after three weeks to non-responders. To motivate participation, a pamphlet describing the study was distributed beforehand to the five limb deficiency centers in Norway. In addition, it was sent to the two Norwegian companies making upper-limb prostheses and assistive devices. The pamphlet was also published on the website of the NLDA.

### Data analysis

Data were entered into a customized database and processed by using the Statistical Package for the Social Sciences (SPSS) version 19.0 (SPSS^®^, IBM, Armonk, NY). For hypothesis testing, the significance level was set at α = 0.05.

To assess the representativeness of our original CLD sample, we used independent samples t-test (for continuous variables) and Fisher’s exact test (for frequencies) to compare the questionnaire responders (n = 97) and non-responders (n = 89). The variables compared were gender, age and place of residence (region). Based on knowledge on general prevalence of CUULD among CLD (two out of three) [3,4], we calculated response rate for CUULD in our sample.

Demographic data were analyzed using descriptive statistics. To compare the extent of pain and fatigue between younger and older adults with CUULD, a cut-off age was set to 40 years, because our clinical experience indicates increased pain and fatigue from that age. Distribution of pain locations in the study sample was compared with reported values from the NGP [19], by using the chi-square test for goodness of fit.

Univariate logistic regression analyses were used to explore the association between the dependent variable chronic pain (yes/no) and several factors: age (≥ 40 years (yes/no), gender, parenthood, living with a partner, comorbidity (additional diagnoses), prosthesis-use (active or passive prosthesis), grip ability, cold sensitivity, education (≥ 13 years, yes/no) and studying or working (without any disability pension), SF-36 physical functioning, SF-36 mental health and FSS. Age, gender and the variables that were significantly associated with chronic pain in the univariate analyses were entered simultaneously into a multivariate logistic regression analysis. The strength of each association was expressed as an odds ratio (OR) with a 95% confidence interval (95% CI).

We used Q-Q plots to assess whether the FSS-scores for the analyzed subgroups were near to a Normal distribution. Linear regression models were used to examine the influence of different factors on fatigue measured with FSS. We considered the following factors: age (≥ 40 years, yes/no), gender, parenthood, living with a partner, comorbidity (additional diagnoses), prosthesis-use (active or passive prosthesis), grip ability, cold sensitivity, education (≥ 13 years, yes/no) and studying or working (without any disability pension), SF-36 physical functioning, SF-36 mental health and chronic pain (yes/no). First, univariate analyses were performed consecutively with FSS as dependent variable, analyzing one independent variable at a time. Second, multiple regression models were applied using the independent variables age, gender, and the variables that were significantly associated with the FSS in the univariate analyses.

## Results

### Response rate and analyses of representativeness

Of 186 available persons with CLD, 97 persons filled in the questionnaires, yielding a 52.2% response rate in the original CLD study [1]. There were no significant differences between the responders and the non-responders regarding place of residence. There were, however, differences regarding mean age (among the responders, 7.6 years higher, p<0.001) and gender (more women among the responders, p = 0.003). Seventy seven of the 97 responders had CUULD. The response rate for CUULD was calculated to approximately 62%.

### Demographic and clinical factors

**Fig 1:**
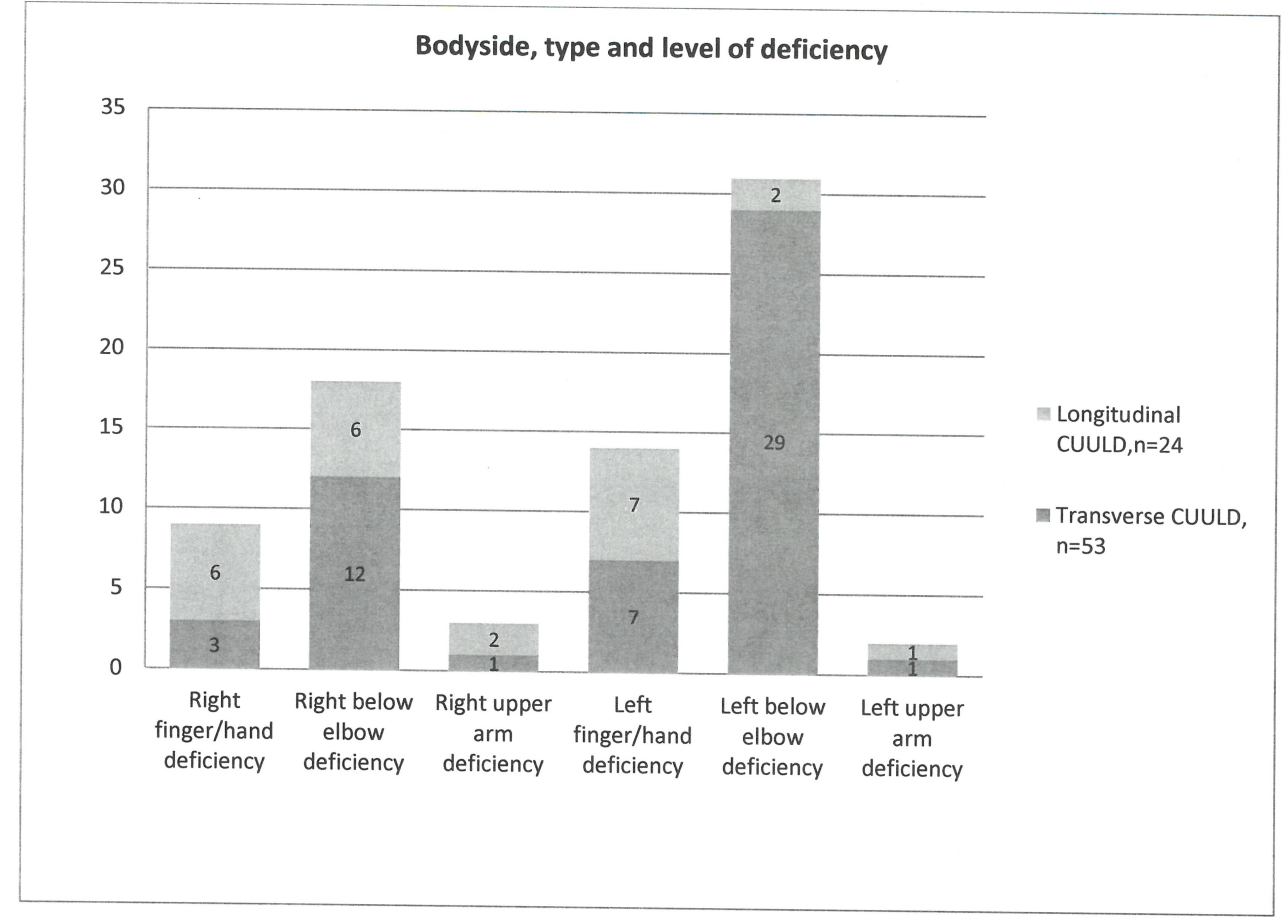
Type, body side and level of the deficiency. Figure shows number of persons with different deficiencies.

The distribution of CUULD types is shown in Fig 1. Most reported left-sided (47/77), below elbow (49/77) deficiency and 53/77 reported transverse deficiency. Demographic and clinical factors are shown in Table 1.

**Table 1:** Demographic and clinical factors for the study group.

Characteristics	Study group (n = 77)
Gender: Women, n (%)	54 (71)
Mean age (SD) (range)	42.7 (16.0)(20–82)
Living with partner, n (%)	52 (68)
Parenthood, n (%)	53 (70)
Formal education >12 years, n (%)	37 (49)
Occupational status	
Studying or working, n (%)^a^	52(68)
Disability-/rehabilitation-/age pension, n (%)^b^	25(32)
Comorbidity, n (%)^c^	34(44)
Cold sensitivity, n (%)^d^	50(65)
Chronic musculoskeletal pain n (%)	49(63)
Grip ability: yes, n (%)^e^	18(23)
Prosthesis user: yes, n (%)^f^	36(47)
SF-36 physical functioning, mean (SD) (range)	84.5 (18.3)(25–100)
SF-36 mental health, mean (SD) (range)	75.0 (18.0)(15–100)

^a^ “Studying or working” include persons who were studying or working full time or part time (without any disability benefit), 43% worked full time.

^b^14% received full disability pension

^c^ Asthma/ allergy, n = 14. Anxiety/ depression, n = 8. Metabolism high/ low, n = 4. Diabetes

n = 3. Psoriasis, n = 2. Arterial fibrillation, n = 2. Blood pressure high/ low, n = 2. Ankylosing spondylitis, n = 1. Schizophrenic, n = 1. Liver disease, n = 2.

^d^ Sensitive to cold.: Tendency to easily freeze on the deficient limb.

^e^ Grip ability: Reduced grip or near to normal grip.

^f^ Prosthesis users: Persons using active or passive prostheses.

### Pain

Chronic musculoskeletal pain was reported by 63%. Among those reporting pain, neck pain (78%) and pain in shoulder /upper arm (78%) was most common, followed by pain in elbow/forearm (57%) and upper back pain (51%). Bilateral pain was common in all pain locations, regardless of body side affected by the deficiency. Persons with unilateral pain tended to have most pain in the same body-side as the deficiency, Table 2.

**Table 2:** Pain locations for persons with CUULD reporting pain (n = 49), contralateral and ipsilateral to the deficiency.

	Pain locations (n = 49)	CUULD left side (n = 30)			CUULD right side (n = 19)		
Pain locations^a^		Pain ipsi-lateral side	Pain contra-lateral side	Bilateral pain	Pain contra-lateral side	Pain ipsi-lateral side	Bilateral pain
Head, n (%)^b^	7	1	1	3	0	0	2
Neck, n (%)	38(78)	6	3	16(53)	1	4	8
Upper back, n (%)	25(51)	5	1	12(40)	2	1	4
Shoulder/upper arm, n (%)	38(78)	6	3	16(53)	2	3	8
Elbow/forearm, n (%)	28(57)	2	2	12(40)	0	4	8
Wrist/hand/fingers, n (%)	21(43)	2	1	9	0	3	6
Lower back, n (%)	22(45)	1	2	10(33)	2	1	6
Chest, n (%)	2	0	1	0	1	0	0
Hips/thigh, n (%)	12(25)	1	0	6	1	1	3
Knee/leg, n (%)	9	0	1	3	1	0	4
Ancles/feet, n (%)	5	0	1	2	0	0	2

^a^Pain locations (0–11) created from the pain drawings.

^b^Percentages only given when n>10.

Compared to the NGP [19], more persons with CUULD reported pain in the neck (78% vs 41%, p≤ 0.001), shoulder/upper arm (78% vs 46%, p≤ 0.001), elbow/forearm (57% vs 21%, p≤ 0.001), wrist/hand/fingers (43% vs 21%, p≤ 0.001) and upper back (51% vs 26%, p≤ 0.001). Pain in the head (14% vs 23%, p = 0.15) lower back (45% vs 52%, p = 0.48) knee/leg (25% vs 38%,p = 0.004) and ancles/feet (10% vs 22%, p = 0.05) were reported less frequently in persons with UULD.

On the NPRS (range 0–10), mean pain intensity (PI) for the whole sample was reported to be 3.3 (SD 2.8). Mean PI score in persons reporting PI >1(n = 55), was 4.7 (SD 2.2). Of 11 possible pain locations (range 0–11), mean number of pain locations (NPL) was 3.0 (SD 2.5) for the whole study sample, and in persons reporting mean NPL >1(n = 58), mean score was 4.0 (SD 2.1), Table 3.

**Table 3:** Pain intensity, number of pain locations and fatigue.

Characteristics	CUULD n = 77
^a^PI (NPRS range: 0–10), mean (SD)	3.3(2.8)
Mild pain (score ≤ 3) n (%)	38(49)
Moderate pain (score ≥4≤6) n (%)	27(35)
Severe pain (score ≥ 7) n (%)	11(14)
^b^NPL (range: 0–11), mean (SD)	3.0(2.5)
Few pain locations (0–3 locations) n (%)	43(56)
Moderate pain locations (4–6 locations) n (%)	29(38)
Many pain locations (7–11 locations) n (%)	5(7)
^c^FSS, (range 1–7), mean (SD)	4.0(1.5)
No fatigue (score ≤ = 4), n (%)	39(51)
Borderline (score >4<5), n (%)	11(14)
Severe fatigue (score ≥ 5), n (%)	25(33)

^a^PI = Pain intensity, assessed with the Numeric Pain Rating Scale (NPRS). PI.

^b^NPL = Number of pain locations: head, neck, upper back, shoulder/upper arm, elbow/fore arm, wrist/hand/fingers, lower. back, chest, hips/thigh, knee/leg, ancles/feet.

^c^FSS = Fatigue Severity Scale.

In persons reporting chronic pain (n = 49); pain limited participation in housekeeping for 23, in leisure activities for 18 and in work-life for 15 persons. Five persons reported that pain started in childhood, 13 in adolescence and 37 in adulthood. Self-reported factors reducing pain were physiotherapy/physical activity and exercise (32), painkillers (16) and adjusted activity and rest (7). Self-reported factors increasing pain were heavy physical work, fine motor- and/or static work (42), stress (6) and rest (3).

In the multiple logistic regression model, chronic pain was associated with female gender (aOR = 5.49, p = 0.025), cold sensitivity (aOR = 4.35, p = 0.035) and fatigue (FSS score) (aOR = 1.88, p = 0.029). These findings indicate that women are about 5.5 times more likely to have chronic pain than men, persons who are sensitive to cold are about 4.4 times more likely to have chronic pain than those without cold sensitivity, and persons with high FSS scores are more likely to have chronic pain than those with low FSS scores, when controlling for each of the other factors in the model. The regression model explained 35.1 to 48. 2% of the variance in chronic pain, Table 4.

**Table 4:** Associations between chronic pain and demographic and clinical factors (n = 77).

Independent variables	Chronic pain			Crude effect estimates*			Adjusted effect estimates**		*p* aOR
	Yes(n)	No(n)	% Yes	cOR	95%CI for cOR	*p* cOR	aOR	95%CI for aOR	
Age, ≥40 years									
Yes	32	10	76	3.39	1.28 to 8.95	0.004	2.74	0.60 to 12.56	0.195
No	17	18	49	Ref	Ref		Ref	Ref	
Gender, women									
Yes	38	16	70	2.59	0.95 to 7.08	0.063	5.49	1.24 to 24.24	0.025
No	11	12	48	Ref	Ref		Ref	Ref	
Parenthood									
Yes	39	15	72	3.38	1.22 to 9.35	0.019	0.64	0.11 to 3.70	0.639
No	10	13	43	Ref	Ref		Ref	Ref	
Living with a partner									
Yes	38	15	72	3.99	1.10 to 8.15	0.032	0.94	0.17 to 5.07	0.936
No	13	11	54	Ref	Ref		Ref	Ref	
Education ≥ 13 years									
Yes	22	15	60	0.76	0.30 to 1.95	0.571	NI		
No	25	13	66	Ref	Ref				
Studying or working									
Yes	31	26	54	0.13	0.03 to 0.63	0.011	0.27	0.02 to 3.26	0.299
No	18	2	90	Ref	Ref		Ref	Ref	
Comorbidity									
Yes	25	9	74	2.17	0.02 to 5.79	0.121	NI		
No	23	18	56	Ref	Ref				
Prosthesis user									
Yes	27	10	73	2.21	0.85 to 5.75	0.104	NI		
No	22	18	55	Ref	Ref				
Grip ability									
Yes	12	6	67	1.19	0.39 to 3.62	0.760	NI		
No	37	22	63	Ref	Ref				
Sensitive to cold									
Yes	37	13	74	3.56	1.33 to 9.55	0.012	4.35	1.11 to 17.13	0.035
No	12	15	44	Ref	Ref		Ref	Ref	
Fatigue Severity Scale^a^				1.77	1.23 to2.56	0.002	1.88	1.07 to 3.31	0.029
SF 36 Physical functioning^a^				0.91	0.86 to 0.97	0.002	0.97	0.91 to 1.04	0.451
SF-36 Mental health^a^				0.99	0.97 to1.02	0.510	NI		

Abbreviations: cOR, Crude odds ratio; aOR, adjusted odds ratio; CI, confidence interval; NI, not included in adjusted model; Ref, reference category.

*Crude effect estimates: logistic regression analysis, one independent variable in the model at a time.

**Adjusted effect estimates: logistic regression analysis, estimates adjusted for the included covariates.

Age, ≥ 40 years: yes = 1, no = 0, Gender, women: yes = 1, no = 0. Parenthood: yes = 1, no = 0, Living with partner: yes = 1,no = 0, Education level ≥13 years: yes = 1, no = 0. Student or working: yes = 1, no = 0. Comorbidity: yes = 1, no = 0. Grip ability: yes = 1, no = 0. Sensitive to cold: yes = 1, no = 0. Prosthesis user: yes = 1, no = 0.

^a^ Continuous variables: FSS: Fatigue severity scale (1–7). SF-36 Physical functioning (0–100). SF-36 Mental health (0–100).

R^2^ = 35.1% to 48.2%.

### Fatigue

Mean FSS score was 4.0 (SD 1.5) for the total study group. Severe fatigue was reported by 25/77, no fatigue by 39/77 and borderline fatigue by 11/77, Table 3.

Table 5 shows the univariate and multivariate linear associations between demographic and clinical factors and the FSS score. In the multivariate analyses, chronic pain (p = 0.016) and mental health (p = 0.046) was found to be significantly associated with fatigue. The regression model explained 22.8% of the variance in the fatigue scores.

**Table 5:** Associations between fatigue and demographic and clinical factors (n = 77).

Independent variables		Linear regression analyses					
	n	cB	95% CI cB	*p* cB	aB	95% CI aB	*p* aB
Age, ≥ 40 years	77	0.55	-0.14 to1.24	0.114	-0.14	-0.96 to 0.68	0.733
Gender, woman	77	-0.13	-0.90 to 0.64	0.736	-0.45	-1.17to 0.27	0.213
Parenthood	76	0.79	0.04 to 1.51	0.038	0.47	-0.38 to 1.33	0.273
Living with a partner	75	0.50	-0.21 to 1.33	0.152	NI		
Education level ≥13 years	76	-0.11	-0.82 to 0.59	0.753	NI		
Studying or working	75	-1.01	-1.82 to -0.23	0.011	0.06	-0.93 to 1.04	0.907
Comorbidity	75	0.12	-0.60 to0.88	0.691	NI		
Prosthesis user	77	0.53	-0.21 to1.23	0.173	NI		
Grip ability	74	-0.97	-0.10 to 0.63	0.644	NI		
Sensitive to cold	75	0.04	-0.71 to 0.84	0.914	NI		
Chronic pain	77	1.20	0.51 to 1.84	0.001	0.90	0.17 to 1.63	0.016
SF 36 Physical functioning	76	-0.03	-0.05 to -0.01	0.001	-0.02	-0.04 to0.01	0.168
SF-36 Mental health	77	-0.03	-0.05 to -0.01	0.003	-0.02	-0.04 to 0.00	0.046

Fatigue measured by Fatigue Severety Scale. A low FSS score indicates low fatigue.

Abbreviations: B unstandardized regression coefficient; cB, crude B; aB, adjusted B- adjusted for age and gender and the variables that were significant in the univariate linear regression; n, number of persons with CUULD available for crude analysis (univariate linear regression). NI, not included in adjusted model

Age, ≥ 40 years: yes = 1, no = 0, Gender, women: yes = 1, no = 0. Parenthood: yes = 1, no = 0, Living with partner: yes = 1,no = 0, Education level ≥13 years: yes = 1, no = 0. Student or working: yes = 1, no = 0. Comorbidity: yes = 1, no = 0. Grip ability: yes = 1, no = 0. Sensitive to cold: yes = 1, no = 0. Prosthesis user: yes = 1, no = 0.

SF-36 physical functioning (0–100). SF-36 mental health (0–100).

R^2^ = 22.8%.

## Discussion

### Main findings

In this study, almost two thirds of persons with CUULD reported chronic pain, many had bilateral pain, and the most common pain locations were neck and shoulder/upper arm. However, pain intensity and the number of pain locations were moderate to low. Many reported that pain started in adult age, and limited participation in everyday life. The study population reported that heavy physical work, static work and stress increased pain, and that physiotherapy, exercise, painkillers, adjusting activity and rest reduced pain. Persons being cold sensitive and persons with high fatigue scores were most likely to have chronic pain. One third reported severe fatigue, the association between pain and fatigue was strong.

### Pain

We have not found previous studies describing pain in adult (≥ 40 years) CUULD populations. In our sample nearly two thirds reported chronic pain. In a Norwegian study on NGP, between 19 and 31% reported chronic pain, varying between age-groups, most pain was reported among the elderly [35]. In persons with CUULD, the most frequent pain locations were neck, shoulder/upper arm, elbow/forearm and upper back, significantly higher than reported in the NGP [19]. In the NGP lower back pain was most frequent [19]. In accordance with our findings, Østlie et al. found a high frequency of neck/ upper back and shoulder pain among acquired UULD [12]. In two mixed study groups of acquired UULD and CUULD, many reported neck/ shoulder and upper back pain and more than one third has been found to have Carpal tunnel syndrome (CTS) [10,13]. In our study sample we found the same fraction reporting pain in hand/fingers. CTS have not been investigated in groups of CUULD.

In our sample, more than 50% reported few pain locations, and 7% reported many pain locations. This is in accordance with findings among acquired UULD [12], but in contrast to Norwegians with chronic pain, were only 20% reported few pain locations and approximately 50% reported many pain locations [19].

We have not found previous reports on pain intensity (PI) among adults with CUULD. In accordance with findings in persons with acquired UULD [12], the majority of our CUULD sample reported mild/moderate PI, and only a small fraction reported severe pain. The mean PI reported by the CUULD group with chronic pain (4.7) was higher than in Norwegians with chronic pain (3.9) [35], and in persons with rheumatoid arthritis (4.3) [36], but slightly lower than in persons with post- polio (5.3) [37].

This might be because persons with a distinct visible disability such as limb loss, do not need to underline their pain, they become believable anyway. This attitude we know from the clinic, and have earlier been described in Johansen et al. [38]. Also, these findings suggest that acquired- and CUULD increases the risk of localized pain, most likely due to overuse and compensative behavior, but perhaps does not increase the risk of generalized pain [10–13]. Results however, have to be interpreted with caution as differences may be due to methodological issues.

The level of PI and NPL are found to have major impact on how the individual lives with pain on a daily basis [35]. Many can live well with some pain, but the more NPL and the higher PI, the more the pain will influence function, activity and participation [35]. In the clinic we meet persons with CUULD reporting pain. Some convey that they do not perceive pain as bothersome in a way that interferes with their life. Others may talk about severe bothersome pain that threaten both everyday life and work-life. Maybe pain has particular importance for some persons with marginal function, like some individuals with CUULD. And maybe, those who struggle to pass as non-disabled [39] are especially vulnerable.

Chronic musculoskeletal pain is often associated with fear [40] and hence it is possible that presence of pain increases fear of losing independence in everyday life. It is conceivable that the individual pain may be experienced as a notification of loss of function and coping, and that pain reporting can be an expression of this. This topic was also underlined in a Norwegian interview study with women with CUULD [38]. The importance of being like everybody else, have also been discussed in relation to other study populations with moderate disability; late onset polio [23] and CP [41]. Most of our CUULD participants reported pain debut in adulthood. This may indicate that pain comes gradually after years with compensatory coping strategies that often are physically demanding.

Factors reported by persons with CUULD to influence pain are well known from our clinical experience and are supported in the literature [18, 42]. Heavy physical work, static work, fine motor activities, and stress will provide overuse and pain over time in all persons [42]; and persons with marginal function as persons with CUULD may be even more vulnerable. Exercise, physical therapy, physical activity, painkillers, and rest are known factors to alleviate pain [18]. Approximately half of the study sample reported physical therapy and physical activity reduced pain. In spite of sparse knowledge about the effect of training and physical exercise on adults with CUULD, it is likely that exercise and necessity of finding new ways of coping may be helpful, as described by Stoelb et al. [36] and Johansen et al. [38]. Adjusted and individual rehabilitation program for persons with CUULD should be developed and the effect on pain should be investigated.

### Fatigue

We have not found previous studies describing fatigue in either acquired UULD or CUULD populations. Mean fatigue (4.0) in the study group was equivalent to that reported in NGP [25]. However, severe fatigue was reported by 33%, considerably higher than in the NGP (22%) [25], reflecting that although the mean fatigue score was low, a significant proportion of adults with CUULD report severe fatigue.

### Pain and fatigue—associations to demographic and clinical factors

In the multivariate analyses age and education level was not associated with pain or fatigue. However, in our study group the mean age was relatively low, and the education level was relatively high, therefore we might have a selection bias. Therefore, these findings have to be interpreted with caution. Female gender was associated with pain but not with fatigue. Some studies show that women are more prone to develop chronic pain [19] and fatigue [25] in the NGP. However, in our study group the proportion of men was low; the result therefore has to be interpreted with caution.

Being cold sensitive was associated with pain when controlling for other factors in the model. To our knowledge, examination of cold sensitivity in persons with CUULD has not been done previously. In persons with hand pathology, cold sensitivity is a common problem [43, 44] and those with severe cold sensitivity report cold-induced pain [44]. In persons with CUULD it is known that blood vessels and nerves may be affected in the side of the deficiency [7]. Reduced blood flow may perhaps cause cold sensitivity and ischemic pain. In the clinic we experience that several persons report bothersome cold sensitivity and that some respond well to warming assistive devices. This is in line with Vaksvik et al.`s findings on persons with hand injury and cold hypersensitivity [43]. It is likely that cold sensitivity, chronic pain and sever fatigue may individually affect function, and may together reinforce functional problems. In persons with CUULD this needs to be investigated further.

Low mental health was significantly associated with high fatigue scores. A strong association between fatigue and mental health is also known from other patient populations [45, 46]. In persons with CUULD, reduced ability to perform daily activities and/or work tasks due to the limb loss, may lead to both mental health challenges and reported fatigue. In accordance with this, it has been shown that reduced ability to work is associated with a tendency to poorer mental health in persons with acquired UULD [47].

In the present study, chronic pain and fatigue were significantly associated, when controlling for other variables. This close relation between fatigue and chronic pain has also been found both in general populations [18, 48] and in other diagnoses with musculoskeletal problems [21–23]. Pain and fatigue may be seen as part of the same phenomenon; as discussed above it is physically demanding over time to manage with one functioning arm; this may challenge the patient’s capacity and cause both pain and fatigue [2].

One might have expected an association between chronic pain/fatigue and limb deficiency variables like prosthesis use and grip ability. We found no such associations, and neither did Østlie et al. in acquired UULD [12]. The multiple regression models explained 35–48% of the variation in chronic pain and 23% of the variation of fatigue implying that other factors are associated with chronic pain and fatigue. These findings need to be explored further.

### Strength and weaknesses

A low response rate is a weakness of our CLD-study. This may have led to selection bias in our sample. The respondents were older, with a lower percentage of men than the non-responder group. This may have affected our findings regarding variables that are known to be gender-dependent, such as pain and fatigue [19, 25]. Including persons with CUULD only from the TRS register, may have resulted in selection bias. Those who are registered at TRS usually have done so because of some need for services, thus potentially affecting some of the variables studied. Persons choosing to register at TRS may also differ from those who do not in relation to socioeconomic factors such as educational level [49].

Self-reported data may be influenced by recall bias. The relatively small sample size leads to reduced statistical power for several analyses, especially regarding differences between subgroups. Using a non-validated cold sensitivity measure may have caused information bias. The survey was conducted late in the fall, a rather cold season in Norway. Pain and cold sensitivity can be affected by cold weather; therefore, we may have an information bias resulting in higher estimates of pain and cold sensitivity than if the study had been conducted in the summer. In Norway, however, it is cold weather during all seasons, and fall is far warmer than the winter time. We therefore estimate this bias as minor. One strength of our study is that our material is recruited from a defined geographical area (Norway) with a defined population (5 million inhabitants), and the response rate among CUULD was considered to be satisfactory and is higher than in our original CLD study [1]. Another strength is that we have studied adults, who have answered questions for themselves about issues that have not been investigated before. Furthermore, pain and fatigue were explored using standardized and validated measurements.

## Conclusion

Many adults with CUULD report chronic pain, often bilateral, primarily located to the neck and shoulder. However, they report moderate to low pain intensity and a low number of pain locations compared to other chronic pain study populations, indicating that a significant portion do not experience the pain as severely bothersome. A significant fraction also report cold sensitivity and severe fatigue. A strong association between pain and fatigue may indicate that everyday life with only one functioning arm is physically challenging, increasing the risk of developing pain and fatigue. These factors may influence functioning and should be taken into account when rehabilitation programs for persons with CUULD are developed.
